# Supplementary material for: Consortium for the Study of Pregnancy Treatments (Co-OPT): An international birth cohort to study the effects of antenatal corticosteroids
Source: PLoS One. 2023 Mar 2;18(3):e0282477. doi: 10.1371/journal.pone.0282477 (PMC9980789; doi:10.1371/journal.pone.0282477)
Supplement: S1 Text — (PDF) [file pone.0282477.s001.pdf]

S1 Text. Data cleaning processes

Identification of births

Inclusion and exclusion criteria (detailed in Methods) were applied to each dataset to determine all births to be included within the Co-OPT ACS cohort. Finland, Israel and Nova Scotia provided maternity and neonatal data as single records, each of which contained all data for one pregnancy and birth, and Iceland provided single records for each infant. Scotland's maternity data was provided from SMR02, which is an episode-based database: an episode is created every time a woman is discharged from obstetric specialties. To identify delivery episodes from SMR02, the variable "CONDITION_ON_DISCHARGE" was used, equating to "3 - Delivered". Furthermore, all births from the same mother were chronologically sorted, and intervals between two adjacent births were calculated to determine the feasibility of births based on the following rules:

1) A birth >154 days from the previous birth was considered a feasible birth from a new pregnancy;
2) A birth within [2, 154] days from the previous birth was considered unfeasible, and thus removed from the cohort;
3) A birth on the next day from a previous **feasible** birth was considered one of the multiple births from the same pregnancy, but any adjacent births on the following day were considered unfeasible and removed; a birth on the next day from a previous **unfeasible** birth was also considered unfeasible and was therefore removed;
4) A birth on the same day from a previous **feasible** birth was considered one of the multiple births from the same pregnancy, otherwise, it was considered unfeasible and removed.

Each SMR02 delivery episode contained births for up to three babies, which were extracted to harmonise with neonatal and CHP datasets.

De-duplication

Each record from Finnish Medical Birth Register and Nova Scotia Atlee Perinatal Database had unique neonatal identifiers, which were used to ensure there was no duplication of records.

The Iceland Medical Birth Register provided unique identifiers at the level of the birth, the child, and the mother. Records with duplicated child identifiers but different birth identifiers or mother identifiers were excluded. For records with duplicated child identifiers which had the same birth identifier or mother identifiers, the most complete record was kept.

Maternal ID and date of birth were used in SMR02 to identify conflicting records if the demographic and clinical data differed between two or more records with the same maternal ID and date of birth. The conflicting records containing the most complete data were retained. Birth data from Israel did not have an accompanying maternal or neonatal identifier, so it was not possible to assess for conflicting records from the Rabin Medical Center Database.

Data outliers, nonsensical values, and data cleaning rules

Descriptive statistics generated for each region were reviewed by Co-OPT collaborators and compared with published research based on the contributing datasets. Collaborators advised whether results were as expected, based on previous analyses and “real-life” clinical experience. Where Co-OPT results were inconsistent with published findings, these were queried directly with data providers, to check data quality. Collaborators created rules for data outliers and nonsensical values, which were applied to the whole cohort, as described below:

- Maternal age at delivery (years): [13 to 60]
- Maternal BMI at booking (kg/m^2): [12 to 90]
- Maternal height* (m): [1 to 2.2]
- Maternal weight*(kg): [35 to 250]

- Parity: [0 to 20]
- Birthweight* (g): [100 – 5500]

*Data cleaning rules for variables highlighted with an asterisk have been adopted from pre-existing Public Health Scotland criteria used to assess data quality [1].

Variables which fell outside of this range were labelled as missing.

Data completeness and harmonisation of datasets

After data cleaning processes were complete, each dataset was reviewed to assess the availability of variables, and data completeness for these variables was evaluated, as shown in Table 3. Where variables were only available for some, but not all, countries, clinical relevance was considered to determine which should be included in secondary analyses, sensitivity analyses and which should be removed from the cohort. This enabled the successful harmonisation of variables across all five datasets, as shown in S1 Table. Some variables were derived from others or defined using ICD-10 codes (as detailed in Tables A-F in S1 File). Further information is available in the Co-OPT ACS data dictionary at https://datashare.ed.ac.uk/handle/10283/4768.

References for S1 Text

1. SMR Datasets: Data Dictionary: SMR02 - Maternity Inpatient and Day Case. Public Health Scotland. [cited 01/07/2022]. Available from: https://www.ndc.scot.nhs.uk/Data-Dictionary/SMR-Datasets//SMR02-Maternity-Inpatient-and-Day-Case/.
