# Supplementary material for: Consortium for the Study of Pregnancy Treatments (Co-OPT): An international birth cohort to study the effects of antenatal corticosteroids
Source: PLoS One. 2023 Mar 2;18(3):e0282477. doi: 10.1371/journal.pone.0282477 (PMC9980789; doi:10.1371/journal.pone.0282477)
Supplement: S2 Text — (PDF) [file pone.0282477.s002.pdf]

S2 Text. Data linkage processes

Scotland

A mother-child lookup table, derived from SMR02 and NRS live births, was provided by eDRIS for linking babies in SBR, identified by child ID and birth order, to their mothers in SMR02, identified by mother ID. Child IDs associated with conflicting dates of birth or mother IDs were removed from the mother-child lookup table before linkage.

Singleton stillbirths in NRS Stillbirths were identified by filtering for records with TOTAL_BIRTHS_LIVE_AND_STILL, LIVE_FEMALES, LIVE_MALES, LIVEBORN_SEX_NOT_KNOWN, STILLBORN_FEMALES, STILLBORN_MALES and STILLBORN_SEX_NOT_KNOWN equating to zero. Due to potential discrepancies between the two datasets, not all of the NRS singleton stillbirths could be linked to SMR02 using mother ID and date of birth. A second attempt at linkage allowed a +/- 2 day difference from the date of birth in NRS, to maximise capture. The SMR02-NRS stillbirths linked using this method were verified by comparing baby sex and birthweight from the two datasets; if neither of these matched, the linkage was considered invalid, and was discarded.

NRS Child Deaths were linked to SMR02 using child ID, birth order and mother ID in the mother-child lookup table.

Based on the birth outcome recorded in SMR02, each baby was assigned a birth outcome using the linked NRS Stillbirths and Child Deaths data as follows:

1. If a baby was present in NRS Stillbirth, s/he was labelled as “NRS Stillbirth”
2. If a baby was present in NRS Child Deaths, his/her lifespan was calculated using the date of birth in SMR02 and the date of death from NRS Child Deaths, from which s/he was labelled as “Early Neonatal Death”, “Late Neonatal Death”, or “Infant Death”
3. If a baby was classified as stillbirth in SMR02, and not present in either NRS Stillbirth or NRS Child Deaths, s/he was labelled as “SMR02 Stillbirth”

4. If a baby was not present in either NRS Stillbirth or NRS Child Deaths, s/he was labelled as "NRS alive"

S1 Fig outlines the filtering, transformation and linkage processes involved in the creation of the linked Scottish dataset within Co-OPT, including SMR02 episodes (which were filtered to include delivery episodes only, and transformed into individual records for each baby), records from NRS Stillbirths, NRS child deaths, and SMR11 and SBR episodes (transformed into individual records for each baby).

Child Health Programme (CHP) records

Review records in CHP follow-up data were linked to babies extracted from SMR02 via child IDs using the aforementioned mother-child lookup table provided by eDRIS.

Aberdeen Maternity and Neonatal Databank (AMND)

The AMND dataset records delivery episodes of mothers in Aberdeen in a similar format to SMR02, but with additional information, such as ACS timing. Each AMND record also contains detailed birth data of up to three babies, which were extracted and linked to babies extracted from SMR02 via child IDs.

Iceland

Hospital Drugs Register

The Icelandic Hospital Drug Register records administration details of all drugs (timing, drug and dose) as one administration per row. This was first filtered for ACS administrations by detecting ATC code "H02AB" (betamethasone and dexamethasone) in the variable "ATC_5_code", before linking with the Icelandic Medical Birth Register extract via mother ID. As Iceland did not provide exact birth dates, there could be up to 31 days error in the ACS-to-birth

interval. Therefore, if any of the ACS administration dates from the Hospital Drugs Register fell within the range [20+0 week gestation age, Birth_month_child + 31 days] extracted from the Icelandic Medical Birth Register extract, or the ATC codes “H02AB” were detected in variable “drugs_mother” from the Icelandic Medical Birth Register extract, the mother was considered to have received ACS in that particular pregnancy. The Icelandic Medical Birth Register was sub-setted for the same period in the Hospital Drug Register before linking the two datasets.

Patient Registers

Icelandic Patient Registers provided contained childhood diagnoses made at hospital and at health clinics.

For hospital records, each row in the Patient Register records one condition (as ICD-10 codes) diagnosed on any day for each child, meaning each child could have multiple rows of records in the Register. For linkage, all conditions of each child were collected on a row organised by date of diagnosis, before linking with the Icelandic Medical Birth Register via child ID.

For health clinic records, the Patient Register lists all child IDs and diagnosis dates in rows, and all conditions diagnosed (as ICD-10 codes) in columns; a tick was put in the corresponding column if a child was diagnosed with a specific condition on a specific date. These records were first filtered for neurodevelopmental conditions (all ICD-10 codes beginning with “F”), then all neurodevelopmental conditions diagnosed for each child were collected on a row, organised by date of diagnoses, and linked with the Icelandic Medical Birth Register via child ID.

Death Register

The Icelandic Death Register records different sections of information on a death certificate on different rows. All death information was collected on the same row for each child before linking with the Icelandic Medical Birth Register via child ID.

Finland

Hospital Register

The Finnish Hospital Register records all out- and inpatient episodes of children. The episode-based datasets were converted to child-based by collecting all episodes of the same children (identified by child encrypted ID) on one row, before linking with the Finnish Medical Birth Register extract via child encrypted ID.
