# Supplementary material for: Consortium for the Study of Pregnancy Treatments (Co-OPT): An international birth cohort to study the effects of antenatal corticosteroids
Source: PLoS One. 2023 Mar 2;18(3):e0282477. doi: 10.1371/journal.pone.0282477 (PMC9980789; doi:10.1371/journal.pone.0282477)
Supplement: S1 Table — ACS = Antenatal corticosteroids. M = Main deciphering variable. C/E = Confounder/Effect Modifier. O = Outcome. √* = available using ICD-10 codes and definitions agreed by Co-OPT collaborators (described in Tables A-F in S1 File). (PDF) [file pone.0282477.s003.pdf]

S1 Table. Key maternity and neonatal variables in Co-OPT ACS cohort available across all contributing datasets after data cleaning and harmonisation

Variable requested	Reason	Finland	Iceland	Israel	Nova Scotia	Scotland
Any ACS administration (yes/no)	M	√	√	√	√	√
Number of births this pregnancy	C/E	√	√	√	√	√
Maternal age at delivery	C/E	√	√	√	√	√
Parity	C/E	√	√	√	√	√
Maternal height at booking	C/E	√	√	√	√	√
Maternal weight at booking	C/E	√	√	√	√	√
Diabetes (pre-existing)	C/E	√*	√*	√	√	√
Hypertension (chronic)	C/E	√*	√*	√	√	√*
Month of birth	C/E	√	√	√	√	√
Year of birth	C/E	√	√	√	√	√
Gestational age at birth	C/E	√	√	√	√	√
Pre-eclampsia	C/E	√*	√*	√	√	√*
Gestational diabetes	C/E	√*	√*	√	√	√
Chorioamnionitis	C/E	√*	√*	√	√	√*
Mode of birth	C/E	√	√*	√	√	√
Onset of labour	C/E	√	√	√	√	√
Stillbirth	O	√	√	√	√	√
Birthweight	O	√	√	√	√	√
Baby sex	C/E	√	√	√	√	√
Congenital anomaly	C/E	√	√*	√	√	√
Apgar score at 5 minutes	O	√	√	√	√	√
Neonatal unit admission	O	√	√	√	√	√

ACS = Antenatal corticosteroids. M = Main deciphering variable. C/E = Confounder/Effect Modifier. O = Outcome. √* = available using ICD-10 codes and definitions agreed by Co-OPT collaborators (described in Tables A-F in S1 File)
