# Supplementary material for: Consortium for the Study of Pregnancy Treatments (Co-OPT): An international birth cohort to study the effects of antenatal corticosteroids
Source: PLoS One. 2023 Mar 2;18(3):e0282477. doi: 10.1371/journal.pone.0282477 (PMC9980789; doi:10.1371/journal.pone.0282477)
Supplement: S2 Table — ACS = Antenatal Corticosteroids. mg = milligrams. NICU = Neonatal Intensive Care Unit. NICE = National Institute for Health and Care Excellence. SOGC = Society of Obstetricians and Gynaecologists of Canada. SMFM = Society for Maternal-Fetal Medicine. MDT = Multidisciplinary Team. RCOG = Royal College of Obstetricians & Gynaecologists. 1 The latest version of the relevant guideline (“Primary source of guidelines”) published during the years when births in the Co-OPT ACS cohort occurred in that region (region-dependent), has been cited for each different source of guidelines, for each region, and changes in guidance over the time studied has been summarised in the table. Please note that some cited guidelines have been archived since publication and have been superseded by updated versions, which were published after the last birth in the Co-OPT ACS cohort from that region occurred. 2 if high risk of imminent medically indicated or spontaneous preterm birth within the subsequent 7 days. √ = routine use of antenatal corticosteroids recommended. (√) = consider use of antenatal corticosteroids. X = routine use of antenatal corticosteroids not recommended. (PDF) [file pone.0282477.s004.pdf]

S2 Table. Overview of key recommendations on use of antenatal corticosteroids across regions included in the Co-OPT ACS cohort

**Region** (Years of births in cohort from region)	**Primary source of guidelines[1]**	**Recommended ACS dosing regimen**	**Standard practice**	**Special considerations**			
			**Administration at 24+0 - 33+6 weeks**	**Administration before 24+0 weeks**	**Administration at 34+0 - 36+6 weeks**	**Administration before planned Caesarean section at 37+0 - 38+6 weeks**	**Use of multiple doses of ACS (rescue or repeat doses)**
**Finland**<br><br>(2006-2018)	The Finnish Medical Society Duodecim and the Finnish Gynecological Association [1]	Two doses 12mg intramuscular betamethasone, 24 hours apart	√ - routine administration recommended[2]	√ - from 2018: **22+5 - 23+6 weeks**: routine administration recommended[2]<br><br>(√) – from 2018: **22+0 – 22+4 weeks** consider at clinician's discretion if newborn care line is active<br><br>√ - from 2014 to 2018: **23+0 - 23+6 weeks**: routine administration recommended[2]<br><br>(√) - from 2010 to 2014: **23+0 – 23+6 weeks**: consider administration[2]	√ - from 2009: **34+0 - 34+6 weeks**: routine administration recommended[2]<br><br>(√) – from 2009: **35+0 – 36+6 weeks**: based on clinical consideration, if suspicion of high risk of neonatal respiratory morbidity, high risk of NICU treatment, or delivery by planned Caesarean section	X – routine administration not recommended	(√) - from 2009: consider repeat course **before 34+6 weeks, if first treatment was given at least 7 days previously**[2]. Routine use of repeat treatments not recommended after 3rd dose.<br><br>X - before 2009: routine use of repeat treatments not recommended
**Iceland**<br><br>(2010-2017)	Regional guidance adapted from National Institute for Health and Care Excellence (NICE) [2]	Two doses 12mg intramuscular dexamethasone, 24 hours apart	√ - routine administration recommended[2]	√ - **22+5 - 23+6 weeks**: routine administration recommended[2]	(√) – in certain cases if delivery by planned Caesarean section	(√) – in certain cases	(√) – consider single repeat dose **if first treatment was before 28 weeks, and was given at least 14 days previously**[2]

**Region** (Years of births in cohort from region)	**Primary source of guidelines**[1]	**Recommended ACS dosing regimen**	**Standard practice**	**Special considerations**			
			**Administration at 24+0 - 33+6 weeks**	**Administration before 24+0 weeks**	**Administration at 34+0 - 36+6 weeks**	**Administration before planned Caesarean section at 37+0 - 38+6 weeks**	**Use of multiple doses of ACS (rescue or repeat doses)**
**Israel** (Rabin Medical Center)<br><br>(2014-2019)	Local adaptation of guidance from American College of Obstetricians & Gynaecologists [3,4]	Two doses 12mg intramuscular betamethasone, 24 hours apart	√ - routine administration recommended[2]	(√) – **23+0 – 23+6 weeks**: consider administration	√ - from 2019: **34+0 - 36+6 weeks**: routine administration recommended, if no previous ACS given[2]	X – routine administration not recommended	(√) – consider single repeat dose **before 34 weeks, if first treatment was given at least 14 days previously** [2]
**Nova Scotia**<br><br>(1990-2018)	Society of Obstetricians and Gynaecologists of Canada (SOGC) [5]<br><br>Society for Maternal-Fetal Medicine (SMFM) [6]	Two doses 12mg intramuscular betamethasone, 24 hours apart (preferred choice)<br><br>or<br><br>Four doses 6mg intramuscular dexamethasone, 12 hours apart	√ - routine administration recommended[2]	**SOGC,** from 2018: (√) - consider after MDT consultation & if early intensive care planned for baby	**SOGC,** from 2018: √ - **34+0 - 34+6 weeks**: routine administration recommended[2]<br><br>**SOGC**, from 2018: (√) – **35+0 – 36+6 weeks:** consider in select clinical situations (routine administration not recommended)<br><br>**SMFM,** from 2016: √ - **34+0 - 36+6 weeks**: routine administration recommended, if high risk of preterm birth before 37 weeks[2]	**SOGC**, from 2018: X – routine administration not recommended	**SOGC**, from 2003: X - routine use of rescue or repeat doses not recommended

**Region** (Years of births in cohort from region)	**Primary source of guidelines[1]**	**Recommended ACS dosing regimen**	**Standard practice**	**Special considerations**			
			**Administration at 24+0 - 33+6 weeks**	**Administration before 24+0 weeks**	**Administration at 34+0 - 36+6 weeks**	**Administration before planned Caesarean section at 37+0 - 38+6 weeks**	**Use of multiple doses of ACS (rescue or repeat doses)**
**Scotland**<br><br>(1997-2018)	Royal College of Obstetricians & Gynaecologists (RCOG) [7]<br><br>National Institute for Health and Care Excellence (NICE) [2]	Two doses 12mg intramuscular betamethasone, 24 hours apart<br><br>or<br><br>Four doses 6mg intramuscular dexamethasone, 12 hours apart	√ - routine administration recommended[2]	**NICE**, from 2015: (√) – **23+0 – 23+6 weeks**: discuss with the woman in context of her individual circumstances	**NICE**, from 2015: (√) – **34+0 – 35+6 weeks**: consider administration[2]<br><br>**RCOG**, from 2010 (archived 2016): √ - **34+0 - 34+6 weeks**: routine administration recommended	**RCOG**, from 2010 (archived 2016): √ - recommended for all women with planned Caesarean **before 38+6 weeks**	**NICE**, from 2015: X – routine repeat courses not recommended

These are general recommendations, which apply only to the time period that births in specific regions in the Co-OPT cohort occurred. Guidance varies within regions in the context of multiple pregnancies and other comorbidities or complications, for example, maternal diabetes or fetal growth restriction.

When guidelines for specific ACS recommendations have been introduced or have changed during the relevant time period, dates have been provided (otherwise, guidelines for that recommendation have been unchanged during the period studied). The table only includes guidelines which recommend, or recommend against, use of ACS within specific patient subgroups (earlier versions of guidelines, which do not include guidance on specific patient populations, have not been included).

ACS = Antenatal Corticosteroids. mg = milligrams. NICU = Neonatal Intensive Care Unit. NICE = National Institute for Health and Care Excellence. SOGC = Society of Obstetricians and Gynaecologists of Canada. SMFM = Society for Maternal-Fetal Medicine. MDT = Multidisciplinary Team. RCOG = Royal College of Obstetricians & Gynaecologists.

[1] The latest version of the relevant guideline (**"Primary source of guidelines"**) published **during the years when births in the Co-OPT ACS cohort occurred in that region** (region-dependent), has been cited for each different source of guidelines, for each region, and changes in guidance over the time studied has been summarised in the table. Please note that some cited guidelines have been archived since publication and have been superseded by updated versions, which were published after the last birth in the Co-OPT ACS cohort from that region occurred.

[2] if high risk of imminent medically indicated or spontaneous preterm birth within the subsequent 7 days

√ = routine use of antenatal corticosteroids recommended. (√) = consider use of antenatal corticosteroids. X = routine use of antenatal corticosteroids not recommended.

References for S2 Table

1. Premature birth: Valid treatment recommendation. Duodecim of the Finnish Medical Society and the Finnish Gynecological Assocation; Käypä Hoito Recommendations; 2018.

2. NICE guideline NG25: Preterm labour and birth. National Institute for Health and Care Excellence; 2015.

3. ACOG Committee on Obstetric Practice: ACOG Committee Opinion No. 713: Antenatal Corticosteroid Therapy for Fetal Maturation. Obstetrics and Gynecology (New York 1953). 2017;130(2):102-9.

4. Position paper no 14: Treatment and prevention of preterm birth, dated 2/10/2013. The Israel society for Maternal-Fetal Medicine and the Israel Union for Obstetrics & Gynaecology; 2013.

5. Skoll A, Boutin A, Bujold E, Burrows J, Crane J, Geary M, et al. No. 364: Antenatal Corticosteroid Therapy for Improving Neonatal Outcomes. Journal of Obstetrics and Gynaecology Canada. 2018;40(9):1291-39.

6. Implementation of the use of antenatal corticosteroids in the late preterm birth period in women at risk for preterm delivery. American journal of obstetrics and gynecology. 2016;215(2):B13-B5.

7. RCOG. Green-top Guideline No. 7: Antenatal corticosteroids to reduce neonatal morbidity and mortality. Royal College of Obstetricians and Gynaecologists; 2010
