# Supplementary figures and images for: Consortium for the Study of Pregnancy Treatments (Co-OPT): An international birth cohort to study the effects of antenatal corticosteroids
Source: PLoS One. 2023 Mar 2;18(3):e0282477. doi: 10.1371/journal.pone.0282477 (PMC9980789; doi:10.1371/journal.pone.0282477)

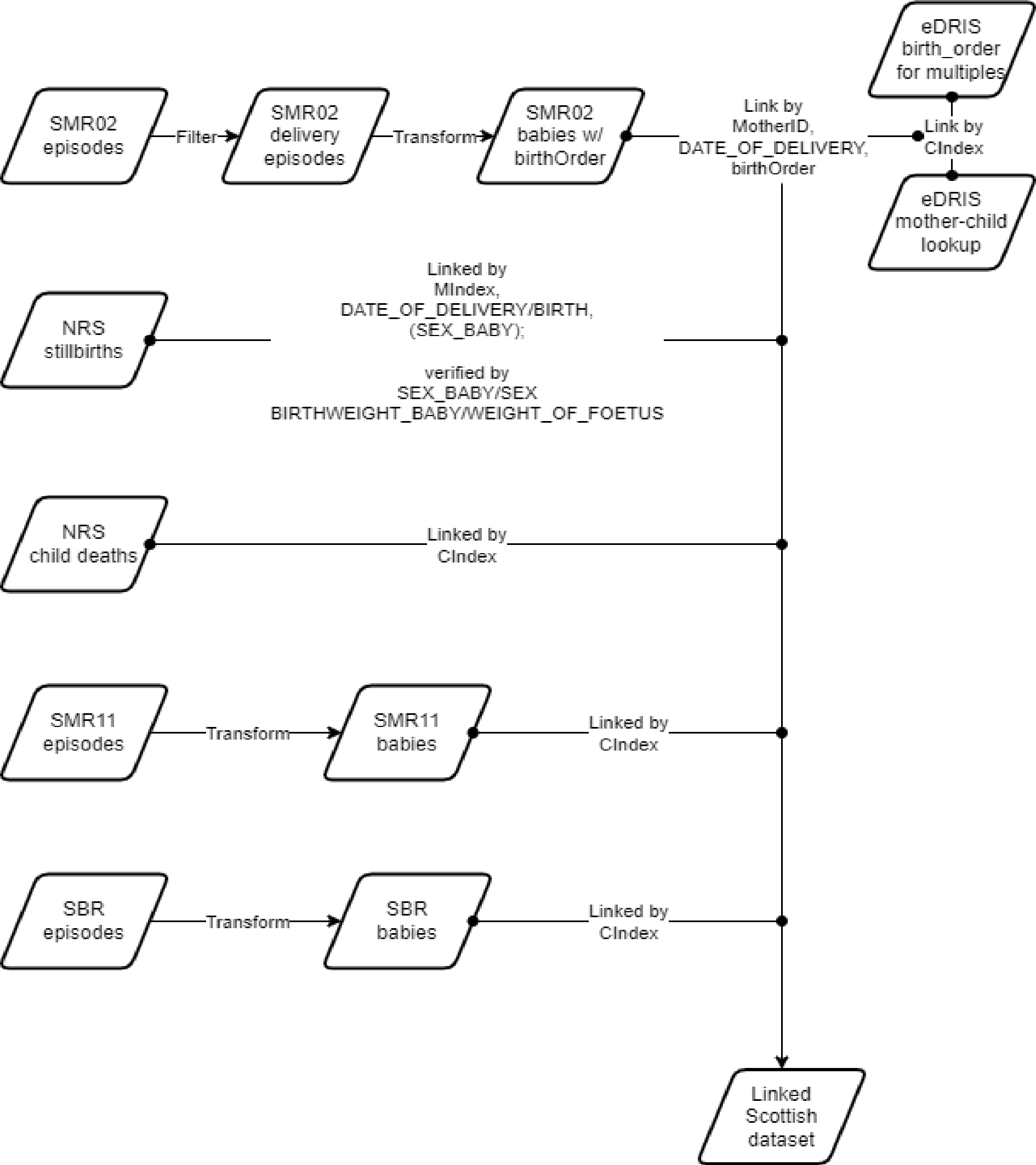

Supplement: S1 Fig — SMR02 = Maternity Inpatient and Day Case Scottish Morbidity Records. eDRIS = electronic Data Research and Innovation Service (Public Health Scotland). MotherID / MIndex = unique mother identifier. CIndex = unique child identifier (child ID). NRS = National Records of Scotland. SMR11 = Neonatal Scottish Morbidity Records. SBR = Scottish Birth Record. (TIF) [file pone.0282477.s005.tif]
