# Supplementary material for: Consortium for the Study of Pregnancy Treatments (Co-OPT): An international birth cohort to study the effects of antenatal corticosteroids
Source: PLoS One. 2023 Mar 2;18(3):e0282477. doi: 10.1371/journal.pone.0282477 (PMC9980789; doi:10.1371/journal.pone.0282477)
Supplement: S1 File — ICD-10 = International Classification of Diseases and Related Health Problems, 10th Revision. (PDF) [file pone.0282477.s006.pdf]

S1 File: ICD-10 code definitions

Tables A-F show ICD-10 (International Classification of Diseases and Related Health Problems, 10th Revision [1]) code definitions, agreed by Co-OPT collaborators, to define maternal and obstetric conditions, congenital anomalies, and mode of birth. For purposes of defining major congenital anomalies (Scotland, Nova Scotia, Israel), the National Health Service (NHS) National Services Scotland Information Services Division amendment to ICD-10 codes provided by EUROCAT (European network of population-based registries for the epidemiological surveillance of congenital anomalies) was used [2].

These ICD-10 code definitions could be applied to any birth register or maternity database which uses ICD-10 codes.

Table A. ICD-10 code definitions for pre-existing maternal diabetes.

Co-OPT variable	ICD-10 search terms	Maternity codes ("O")	Fetal / Paediatric codes ("P")	General codes	Code definition	Include? (Y = Yes, N = No)	Reason for exclusion
**Pre-existing maternal diabetes**	"diab", "diabetes", "mellitus",	O24			Diabetes mellitus in pregnancy: Pre-existing diabetes, non-insulin-dependent	**Y**	/
		O24.0			Diabetes mellitus in pregnancy: Pre-existing type 1 diabetes	**Y**	/
		O24.1			Diabetes mellitus in pregnancy: Pre-existing type 2 diabetes mellitus	**Y**	/
		O24.2			Diabetes mellitus in pregnancy: Pre-existing malnutrition-related diabetes	**Y**	/
		O24.3			Diabetes mellitus in pregnancy: Pre-existing, unspecified	**Y**	/
		O24.9			Diabetes mellitus in pregnancy: unspecified	N	Could be pre-existing or gestational
		O99.2			Endocrine, nutritional and metabolic diseases complicating pregnancy, childbirth and puerperium	N	Too broad – does not specify diabetes mellitus
			P70.1		Syndrome of an infant with a diabetic mother	N	Could be pre-existing or gestational
				Z13.1	Special screening examination for diabetes mellitus	N	Could be screening for gestational diabetes (or diabetic retinopathy)
				E10.x (x = 0-9)	Type 1 diabetes mellitus	**Y**	/
				E11.x (x = 0-9)	Type 2 diabetes mellitus	**Y**	/
				E12.x (x = 0-9)	Malnutrition-related diabetes mellitus	**Y**	/
				E13.x (x = 0-9)	Other specified diabetes mellitus	**Y**	/
				E14.x (x = 0-9)	Unspecified diabetes mellitus	**Y**	/

Table B. ICD-10 code definitions for pre-existing maternal hypertension.

Co-OPT variable	ICD-10 search terms	Maternity codes ("O")	General codes	Code definition	Include? (Y = Yes, N = No)	Reason for exclusion
**Chronic hypertension**	"hypertension", "hypertensive", "high blood pressure"	O10.x (x=0-4, 9)		Pre-existing hypertension complicating pregnancy, childbirth and the puerperium	**Y**	/
		O11		Pre-eclampsia superimposed on chronic hypertension	N	Overlap with pre-eclampsia
		O16		Unspecified maternal hypertension	N	Could be pre-existing or pregnancy-induced
			I10	Essential (primary) hypertension	**Y**	/
			I11.x (x = 0, 9)	Hypertensive heart disease (+/- congestive heart failure)	**Y**	/
			I12.x (x = 0, 9)	Hypertensive renal disease (+/- renal failure	**Y**	/
			I13.x (x = 0-2,9)	Hypertensive heart and renal disease	**Y**	/
			I15.x (x = 0-2,8,9)	Secondary hypertension	**Y**	/

Table C. ICD-10 code definitions for gestational diabetes.

Co-OPT variable	ICD-10 search terms	Maternity codes ("O")	Fetal / Paediatric codes ("P")	Code definition	Include? (Y = Yes, N = No)	Reason for exclusion
**Gestational diabetes**	"gestational diabetes", "diabetes pregnancy"	O24.4		Diabetes mellitus arising in pregnancy	**Y**	/
		O24.9		Diabetes mellitus in pregnancy: unspecified	N	Could be pre-existing or gestational diabetes
			P70.0	Syndrome of infant of mother with gestational diabetes	**Y**	/

Table D. ICD-10 code definitions for gestational hypertension / pre-eclampsia.

Co-OPT variable	ICD-10 search terms	Maternity codes (“O”)	Code definition	Include? (Y = Yes, N = No)	Reason for exclusion
**Gestational hypertension / Pre-eclampsia**	“pre-eclampsia”, “pregnancy induced hypertension”, “eclampsia”, “HELLP”	O11	Pre-eclamspia superimposed on chronic hypertension	**Y**	/
		O12.x (x=0-2)	Gestational oedema and proteinuria without hypertension	N	Does not include hypertension – could be aetiologies distinct from pre-eclampsia
		O13	Gestational [pregnancy-induced] hypertension	**Y**	/
		O14.x (x=0-2,9)	Pre-eclampsia	**Y**	/
		O15.x (x=0-2,9)	Eclampsia	**Y**	/
		O16	Unspecified maternal hypertension	N	Could be pre-existing or pregnancy-induced

Table E. ICD-10 code definitions for chorioamnionitis.

Co-OPT variable	ICD-10 search terms	Maternity codes (“O”)	Fetal / Paediatric codes (“P”)	Code definition	Include? (Y = Yes, N = No)
**Chorioamnionitis**	“chorio”, “chorioamnionitis”	O41.1		Infection of amniotic sac and membranes	Y
			P02.7	Fetus and newborn affected by chorioamnionitis	Y

Table F. ICD-10 code definitions for mode of birth categories.

Co-OPT variable	Categories	Maternity codes ("O")	Code definition
**Mode of birth**	Vaginal	O75.7	Vaginal delivery following previous Caesarean section
		O80.x (x=0,1,8,9)	Single spontaneous delivery
		O83.0	Breech extraction
		O83.1	Other assisted breech delivery
		O83.2	Other manipulation-assisted delivery
		O84.0	Multiple delivery, all spontaneous
	Assisted vaginal	O81.x (x=0-5)	Single delivery by forceps and vacuum extractor
		O83.8	Other specified assisted single delivery
		O83.9	Assisted single delivery, unspecified
		O84.1	Multiple delivery, all by forceps and vacuum extraction
	Planned Caesarean section	082.0	Delivery by elective Caesarean section
	Unscheduled Caesarean section	O82.1	Delivery by emergency Caesarean section
	Other / Unspecified	O83.3	Delivery of viable fetus in abdominal pregnancy
		O84.8	Other multiple delivery by combination of methods

ICD-10 = International Classification of Diseases and Related Health Problems, 10th Revision

References for S1 File:

1. WHO. ICD-10: International statistical classification of diseases and related health problems - 10th revision, Volume 2, Fifth edition: World Health Organization; 2016.

2. Congenital anomalies in Scotland: 2012 to 2017. ISD Scotland, NHS National Services Scotland 26 November 2019. Available from: https://www.isdscotland.org/Health-Topics/Maternity-and-Births/Publications/2019-11-26/2019-11-26-Congenital-Anomalies-in-Scotland-2017-Report.pdf?57386416197.
