# Supplementary material for: Consortium for the Study of Pregnancy Treatments (Co-OPT): An international birth cohort to study the effects of antenatal corticosteroids
Source: PLoS One. 2023 Mar 2;18(3):e0282477. doi: 10.1371/journal.pone.0282477 (PMC9980789; doi:10.1371/journal.pone.0282477)
Supplement: Attachment — Submitted filename: Review_PONE_2022_Frier.docx [file pone.0282477.s007.docx]

**To the authors:**

This paper describes the first study within Co-OPT focusing on antenatal corticosteroids (ACS) in five countries between 1990-2019, including more than 2 million births. The overall strengths include the large cohort to study several important and rare outcomes including perinatal mortality and also neurodevelopmental disorders later in childhood. The limitations with the overall Co-OPT ACS cohort, include large missing numbers for certain exposures, outcomes and covariates, however these are well-described in the discussion section. Nevertheless, this is something the authors will have to take into consideration for each specific subsequent study question using this data.

I believe that this manuscript may be of interest for the readers of PONE and useful in the future for subsequent papers using data from the Co-OPT, but I have some suggestions to improve the manuscript:

1. While the introduction gives a very comprehensive overview of the history of ACS and the knowledge gap, it becomes quite long and some parts may be shortened to keep the interest of the reader.
2. In the methods on page 11, the authors state that the clinical guidelines on use of ACS in the included countries are similar and with guidance from the WHO. The clinical guidelines could be summarized and described with similarities and differences in the appendix, and further acknowledged in the discussion.
3. In the methods on page 12, there is a referral to a URL for a dictionary. However, this URL is not included in the paper and now says “tbc”.
4. Throughout the paper the word “episode” is used. For what I understand, an episode corresponds to a hospital visit or a duration of time including when the woman gave birth or similar. Are you taking the number of days into consideration for these episodes, or is this word used to describe any visits within the health care system? Episode-level data is also mentioned in table 1, which to me is a bit unclear what it means.
5. On page 15, the author states that assessments undertaken to evaluate children’s health, growth etc are done by “health visitors”. Please define what a “health visitor” is.
6. To me it seems unclear that you could have as many pregnancies as births when you also include multiple births. It would be clearer if you described the number of pregnant individuals leading to x number of births. I assume that the problem is that the number of pregnant women in Israel was unknown. This should be acknowledged in the discussion.
7. In the results section on page 28, I would strongly suggest to add that only 15.5% of the whole cohort had available information on timing of ACS exposure.
8. In the discussion section on page 30 the authors discuss that varying rates of ACS exposure in the different countries “likely reflect variations in ACS prescribing practice”. To me this contradicts the previous statement that clinical practice is similar in the different countries. Please clarify both the clinical guidelines (as mentioned in point 2) and in the discussion how potential differences between the countries in prescribing patterns may affect future studies using data on ACS from the full cohort.
