# Supplementary material for: Consortium for the Study of Pregnancy Treatments (Co-OPT): An international birth cohort to study the effects of antenatal corticosteroids
Source: PLoS One. 2023 Mar 2;18(3):e0282477. doi: 10.1371/journal.pone.0282477 (PMC9980789; doi:10.1371/journal.pone.0282477)
Supplement: Attachment — Submitted filename: Response to Reviewers.docx [file pone.0282477.s009.docx]

**Consortium for the Study of Pregnancy Treatments (Co-OPT): An international birth cohort to study the effects of antenatal corticosteroids (PONE-D-22-23203R2) for PLOS One**

**Response to the Academic Editor**

**Comments from the Academic Editor:**

We appreciate your revisions and assert that the manuscript is close to being accepted. We would like you to further qualify the manuscript by outlining the limitations of the dataset given the variation in outcomes. In particular, it is yet to be known that the combined dataset can disentangle time dependent effects of ANC from the multiple co-variates including prematurity related consequences due to missing of essential data and the heterogeneity of contributing institutions with their different focus on infant developmental endpoints. If the combined dataset does not have comparable endpoints of neurodevelopment, it may be more informative to perform single center studies to prevent problems of heterogeneity. Please go through the manuscript to adjust claims that are yet to be determined.

**Thank you for this very helpful guidance. We have added statements to elaborate on the limitations of the dataset, firstly in relation to the impact of missing data and our plans to evaluate this in future analyses (page 36):**

***“The impact of missing data on the robustness of conclusions drawn from the Co-OPT ACS cohort will be carefully evaluated in attrition analyses.”***

**We have also added a statement to highlight the challenges posed by lack of data specifically on the timing of ACS, in the paragraph which begins “Another limitation of the Co-OPT ACS cohort is the lack of information available on either gestational age at exposure to ACS, or on time intervals between ACS administration and birth” (page 36):**

***“Missing data on the timing of ACS exposure increases likelihood of bias from residual confounding, as the effects of ACS exposure are intricately linked with the timing of ACS administration.”***

**We agree that it is more informative to undertake single centre analyses for the associations of ACS exposure with neurodevelopment, in view of the variations in neurodevelopmental outcomes between contributing datasets, and we have added sentences on page 37 to outline this and our approach to addressing this:**

***“Furthermore, given the heterogeneity of neurodevelopmental outcome data from these countries, ongoing analyses of possible associations of ACS exposure with early child neurodevelopment are limited to single centre analyses of data from Scotland alone. The contribution of data from different regions in Co-OPT to analyses of long-term outcomes will vary based on the availability and comparability of longitudinal data available for each outcome”.***

**We have also modified the sentence on page 37 to highlight the fact that heterogeneity of outcomes is a study limitation:**

***“However, the variation in follow-up data between regions is a study limitation, as this reduces the potential to harmonise outcomes across datasets in the cohort.”***

**Finally, the statement already included in the conclusion (page 38) *“the large scale and international representation of the Co-OPT ACS cohort will enable robust assessment of ACS exposure on rare outcomes, such as perinatal mortality”* should clarify that a single centre approach will not be required for all outcomes in the dataset, for example, perinatal mortality.**

**Amendments which ensure compliance with Submission Guidelines**

**Journal requirements:**

Please review your reference list to ensure that it is complete and correct. If you have cited papers that have been retracted, please include the rationale for doing so in the manuscript text, or remove these references and replace them with relevant current references. Any changes to the reference list should be mentioned in the rebuttal letter that accompanies your revised manuscript. If you need to cite a retracted article, indicate the article’s retracted status in the References list and also include a citation and full reference for the retraction notice.

**As we had cited a guideline which has since been archived (on page 32), we have removed this reference, instead providing the relevant information in the manuscript on the archived guideline, and have included reference which cites the current guideline (which has replaced the archived version). We have deleted the reference entirely from both the marked-up version of the manuscript and the unmarked version of the revised paper, to ensure that all references have updated correctly on the reference manager and are accurate. We have reviewed the reference list and can confirm it is complete and correct.**

We note that the grant information you provided in the ‘Funding Information’ and ‘Financial Disclosure’ sections do not match.

When you resubmit, please ensure that you provide the updated Funding Information.

**In keeping with our first revision of the manuscript (PONE-D-22-23203R2), we have addressed this by ensuring that the grant information provided is accurate, and that both grant numbers match: we noted that the Funding Reference number for this study has been abbreviated from 209560/Z/17/A14/32 to 209560/Z/17 in some contexts, for example, in the published protocol (cited in the Methods), so to maintain consistency we have ensured that the abbreviated grant number is used throughout.**

**As detailed in our Cover letter, the updated Financial Disclosure statement should read as follows:**

***The Co-OPT ACS study is funded through a Wellcome Trust Clinical Career Development Fellowship grant (Funding Reference number 209560/Z/17) awarded to Sarah J Stock. The funders had no role in study design, data collection, data analysis, decision to publish, or preparation of the manuscript. The Sponsor of the study is the University of Edinburgh (***[***www.ed.ac.uk***](http://www.ed.ac.uk)***), Sponsor reference AC19119. For the purpose of open access, the author has applied a CC BY public copyright licence to any Author Accepted Manuscript version arising from this submission.***

**Similarly, in keeping with our first revision of the manuscript, we wish to amend the “Competing interests” section, which should read as follows (also included in the Cover letter): *Sarah J Stock is funded by a Wellcome Trust Clinical Career Development Fellowship* *(209560/Z/17) for research into antenatal corticosteroids. The funders had no role in study design, data collection and analysis, decision to publish, or preparation of the manuscript. All other authors have declared that no competing interests exist.***

Thank you for stating the following financial disclosure:
 [The Co-OPT ACS study is funded through a Wellcome Trust Clinical Career Development Fellowship grant (Funding Reference number 209560/Z/17/Z14/32) awarded to Dr Sarah J Stock. The funders had no role in study design, data collection, data analysis, decision to publish or preparation of the manuscript. The Sponsor of the study is the University of Edinburgh ([www.ed.ac.uk](http://www.ed.ac.uk/)), Sponsor reference AC19119. For the purpose of open access, the author has applied a CC BY public copyright licence to any Author Accepted Manuscript version arising from this submission.]

Please state what role the funders took in the study.  If the funders had no role, please state: "The funders had no role in study design, data collection and analysis, decision to publish, or preparation of the manuscript."
If this statement is not correct you must amend it as needed.
Please include this amended Role of Funder statement in your cover letter; we will change the online submission form on your behalf.

**We have addressed this as described in the response above, which includes the updated Financial Disclosure statement and Competing Interests Section, both of which state the role of the funders in the study.**
